# Case Report: Complex cardiac arrhythmia management in the ICU for an adolescent with Friedreich ataxia

**DOI:** 10.3389/fped.2025.1542513

**Published:** 2025-04-29

**Authors:** Oluwatomini A. Fashina, Stephen J. Gleich, Derek N. Opp, Yves Ouellette, Yu Kawai

**Affiliations:** ^1^ Department of Pediatrics Medicine, Mayo Clinic Children's, Rochester, MN, United States; ^2^ Department of Anesthesiology and Perioperative Medicine, Mayo Clinic, Rochester, MN, United States; ^3^ Division of Pediatric Critical Care, Mayo Clinic Children's, Rochester, MN, United States; ^4^ Division of Pediatric Cardiology, Mayo Clinic Children's, Rochester, MN, United States

**Keywords:** arrhythmia, ICU, Friedreich, ataxia, ECMO, pediatric

## Abstract

**Background:**

Friedreich ataxia (FRDA) is a progressive neurodegenerative disorder with specific clinical manifestations, such as scoliosis, which may impact the management of cardiac arrhythmias and heart failure.

**Case description:**

A 17-year-old male with FRDA and hypertrophic cardiomyopathy presented with atrial flutter and resultant acute-on-chronic systolic and diastolic heart failure with reduced ejection fraction. The arrhythmias were refractory to medical management with adenosine and amiodarone. An attempted cavotricuspid isthmus ablation was unsuccessful due to abnormal cardiac positioning caused by severe scoliosis. Despite optimization with dofetilide and metoprolol, he was readmitted with recurrent atrial arrhythmias and cardiogenic shock, secondary to probable amiodarone-induced thyrotoxicosis, requiring extracorporeal membrane oxygenation. His clinical course involved multisystem complications, prolonged hospitalization, and disease progression, with no recovery in systolic function despite control of his arrhythmia burden.

**Conclusion:**

Intensivists should be cognizant of multisystem complications that can arise when treating refractory cardiac arrhythmias, especially in those with concomitant genetic conditions.

## Introduction

Friedreich ataxia (FRDA) is a progressive neurodegenerative disorder, with autosomal recessive inheritance, caused by a guanine–adenine–adenine (GAA) repeat expansion in the frataxin gene on chromosome 9q13 (1–3). FRDA is a multisystem condition characterized by progressive cerebellar ataxia affecting all four extremities, dysarthria, absent deep tendon reflexes in the lower limbs, pyramidal weakness, cardiomyopathy, kyphoscoliosis, and diabetes mellitus (1, 3). Disease onset typically occurs during adolescence and is inversely related to both disease severity and the length of the GAA repeat expansion (3–5). Similarly, an earlier age of onset and a longer GAA repeat expansion are associated with a faster rate of disease progression (3–5).

Scoliosis and cardiomyopathy are both highly prevalent manifestations of FRDA, with scoliosis often being the first non-neurological symptom and cardiomyopathy the leading cause of death (6, 7). Case series report that scoliosis affects >50% of individuals with FRDA and frequently requires surgical intervention (3, 8, 9). In patients with early-onset (0–7 years) and typical-onset (8–14 years) disease, scoliosis prevalence can be as high as 90%, with surgical intervention required in 55% and 25% of cases, respectively (6). Beyond improving posture and quality of life, surgical intervention can potentially mitigate long-term cardiopulmonary dysfunction (6). Cardiac complications, particularly hypertrophic cardiomyopathy, affect approximately 80% of all FRDA patients (10). Although electrocardiogram and echocardiogram abnormalities are common, global systolic function is typically preserved until late in the disease course, with atrial arrhythmias and heart failure being the primary causes of death (10–12). While established treatment strategies focus on heart rate (HR) and rhythm control, there is limited literature on how other clinical features of FRDA impact arrhythmia management (1, 11, 12).

Herein, we describe the unique case of a 17-year-old male patient with FRDA and hypertrophic cardiomyopathy, who experienced a prolonged hospitalization due to complex cardiac arrhythmias. His pediatric intensive care unit (PICU) course was complicated by atrial arrhythmias that were refractory to medical and procedural interventions due, in part, to the atypical anatomical positioning caused by his severe scoliosis. This unique case informs intensivists and cardiologists alike on the impact of genetic conditions on cardiac arrhythmia management and the multisystem complications that might follow.

## Case description

We describe two hospital admissions for refractory atrial arrhythmias and cardiogenic shock involving a 17-year-old male patient with FRDA and hypertrophic cardiomyopathy.

The patient had a heterozygous GAA repeat expansion of 900, with disease onset at age 4 and wheelchair dependence by age 10. Due to limitations in gene sequencing technology, a second mutation could not be identified. However, the patient's clinical findings, including an extremely low frataxin level of 2 (normal ≥19), a similarly affected sibling being homozygous, and both parents being heterozygous, supported a clinical diagnosis of Friedreich ataxia (FRDA).

**Figure 1:**
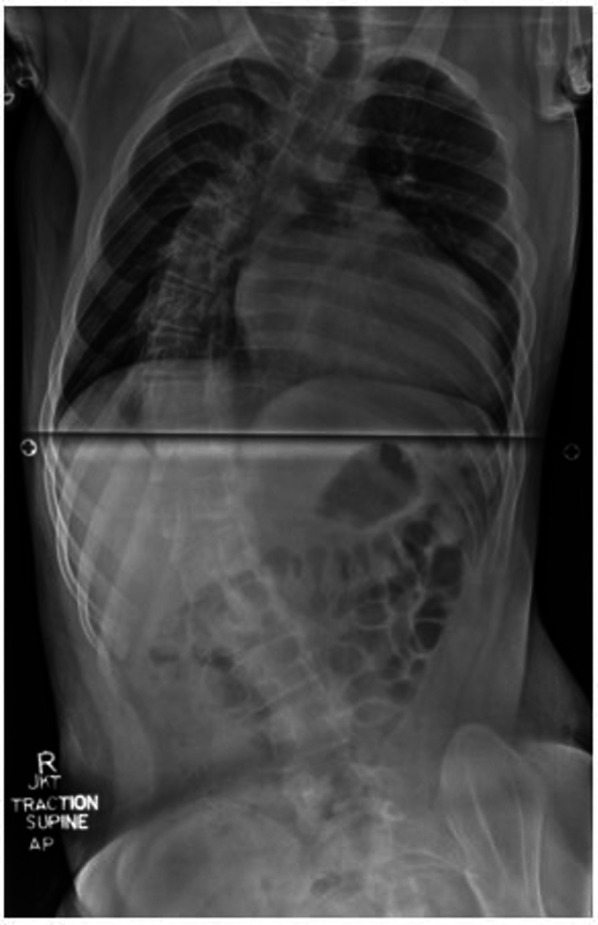
Full spine X-ray demonstrating severe long segment right thoracolumbar rotatory scoliosis.

He had a history of rotatory neuromuscular scoliosis, with a Cobb angle of 130° when sitting and 65° when corrected with traction (Figure 1), classified as severe (angle >40°) (6). Surgical correction was attempted via posterior spinal fusion but was deferred intraoperatively due to a high likelihood of neurological compromise. The patient was diagnosed with cardiomyopathy at age 6 and initially managed with propranolol and verapamil. Two years prior to this current presentation, the patient was hospitalized for the first time with atrial flutter and a reduced left ventricular ejection fraction (LVEF) of 15%–20%. He subsequently required cardioversion and was initiated on oral amiodarone therapy (200 mg daily), with subsequent recovery of LVEF to 45%–59%. Additionally, he had been initiated and maintained on omaveloxolone, a novel therapeutic drug approved by the Food and Drug Administration in February 2023 for the treatment of FRDA, for 3 months prior to admission (13).

### Hospital course 1

**Figure 2:**
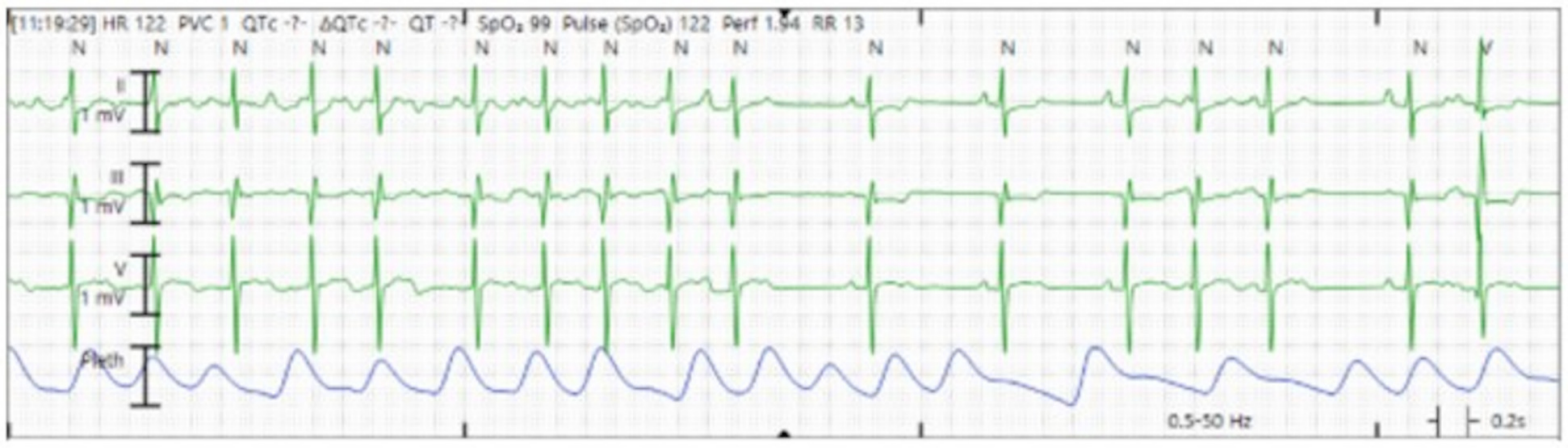
Cardiac telemetry showing conversion from atrial flutter to sinus rhythm, with some atrial ectopy.

Upon initial presentation to the emergency department, the patient was found to have atrial flutter on electrocardiogram (ECG) with an HR of 180–190 beats per minute (bpm). He remained hemodynamically acceptable with a blood pressure of 103/55 mm of mercury. Due to persistent atrial flutter, an adenosine bolus (6 mg) produced a brief decrease in HR to 120–130 bpm with disorganized atrial conduction and prompt return of atrial flutter. He received a loading dose of 150 mg of amiodarone followed by a continuous amiodarone infusion of 1 mg/min for the first 6 h and subsequently 0.5 mg/min. Following this, the patient converted to normal sinus rhythm with some atrial ectopy and was admitted to the PICU for cardiac telemetry monitoring (Figure 2). His amiodarone infusion was discontinued after 24 h, and his oral maintenance dose was increased to 200 mg twice daily (BID). After this bout of atrial flutter, his echocardiogram showed a depressed LVEF of 45%, compared with 56% 1 week prior.

Subsequently, status post amiodarone infusion, his atrial flutter recurred [with variable atrioventricular (AV) conduction], and he was found to have hyperthyroidism, secondary to amiodarone toxicity. Oral amiodarone was discontinued, and oral steroids were started. A cavotricuspid isthmus (CTI) ablation was attempted. The procedure was unsuccessful with easily inducible atrial tachycardia with burst atrial pacing post-ablation. Unfortunately, the procedure was technically difficult due to the patient's severe scoliosis and contractures, which caused significant anatomical distortion and abnormal cardiac positioning. The procedure began with the placement of multipolar catheters via right and left femoral venous access into the coronary sinus and high right atrium. The bundle of His was tagged, and an activation map of the atrial flutter, along with an anatomical shell of the right atrium, was created. Entrainment from the CTI was attempted but resulted in degeneration into atrial flutter. CTI ablation was initiated at the tricuspid annulus and extended toward the inferior vena cava. However, during ablation, the atrial flutter changed in both activation pattern and cycle length. A subsequent attempt at entrainment accelerated the flutter and was uninterpretable. Due to these challenges, the procedure was aborted, and the patient was electrically cardioverted back to sinus rhythm.

In the PICU, he soon returned to atrial flutter. Amiodarone levels returned subtherapeutic without a washout period. Metoprolol tartrate and dofetilide were started and uptitrated, 50 mg BID and 500 micrograms (mcg) BID, respectively, for rate and rhythm control. However, his clinical course was complicated by episodes of nonsustained ventricular tachycardia, likely due to dofetilide. Upon dofetilide downtitration to 250 mcg BID, he developed persistent atrial flutter leading to acute systolic heart failure (LVEF 29%) and, thereafter, acute kidney injury. Eventually, the patient stabilized on metoprolol 50 mg BID and dofetilide 250 mcg every 8 h, converted to sinus rhythm, and was discharged home after demonstration of recovered systolic function (LVEF 44%) and renal function.

### Hospital course 2

**Figure 3:**
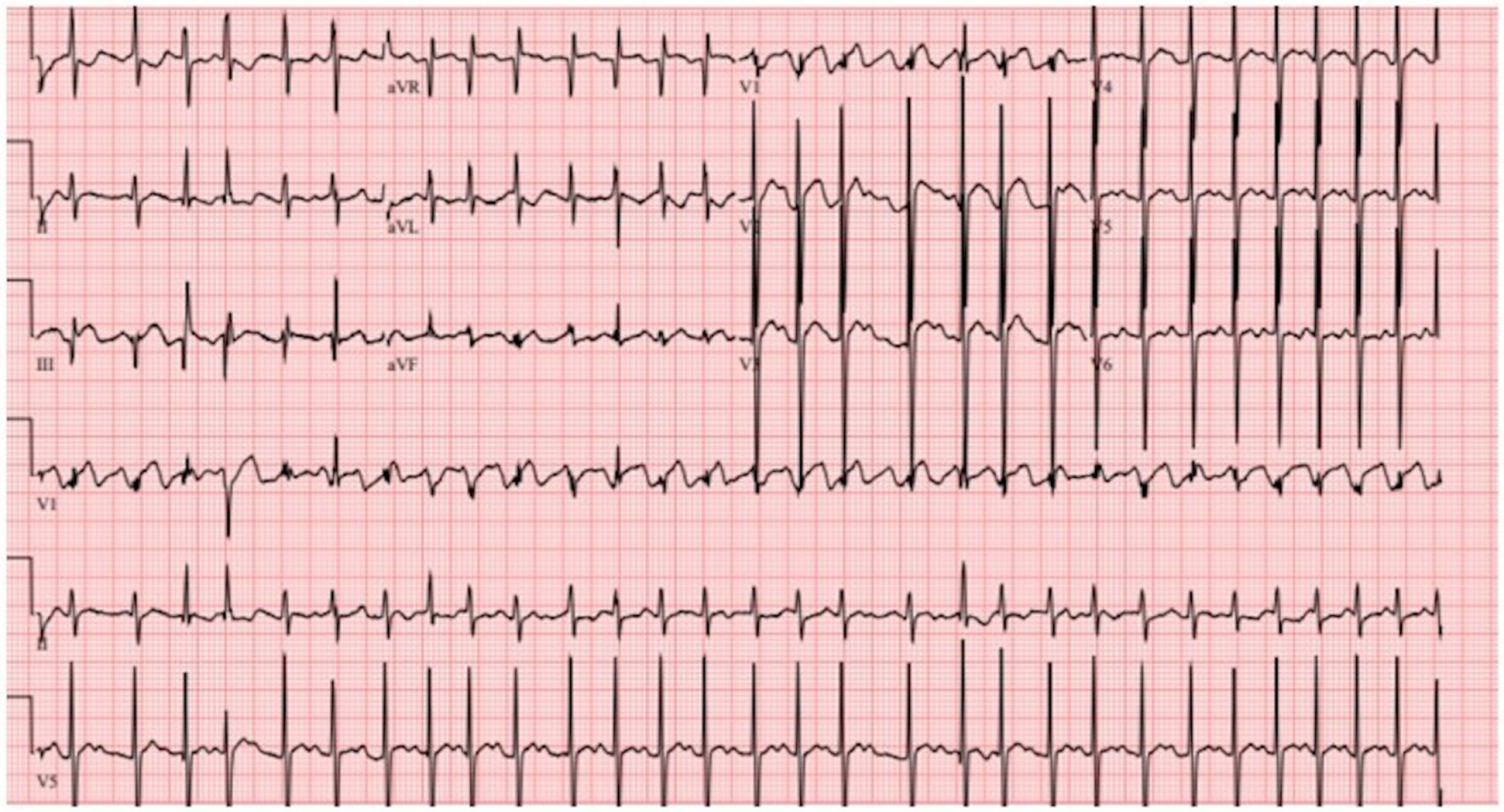
Electrocardiogram showing atrial flutter with rapid ventricular response.

Five days later, the patient was readmitted in critical condition with decompensated cardiogenic shock (LVEF 11%) secondary to hemodynamically unstable atrial dysrhythmia. The initial ECG showed atrial flutter with a rapid ventricular response of 178 bpm (Figure 3), which was refractory to three attempts at cardioversion. Amiodarone load (300 mg) was administered. Due to progressive hemodynamic instability, he was intubated and peripherally cannulated for venoarterial extracorporeal membrane oxygenation (VA ECMO). ECMO cannulation was complicated by left femoral arterial injury, which required surgical repair, that led to ECMO flow cessation and 5 min cardiac arrest due to pulseless electrical activity. ECMO support was reinitiated through cannulation of the right femoral artery.

Once stabilized on ECMO, broad differential diagnoses were considered for his presumed increased catecholamine state that resulted in recurrent unstable atrial arrhythmia and cardiogenic shock. Ultimately, amiodarone-induced thyrotoxicosis (AIT) type 2 (occurring in patients without underlying thyroid disease), exacerbating his tachyarrhythmias, was diagnosed. This was refractory to prednisone, methimazole, high-dose propylthiouracil, and plasmapheresis. He required a thyroidectomy for definitive management.

**Figure 4:**
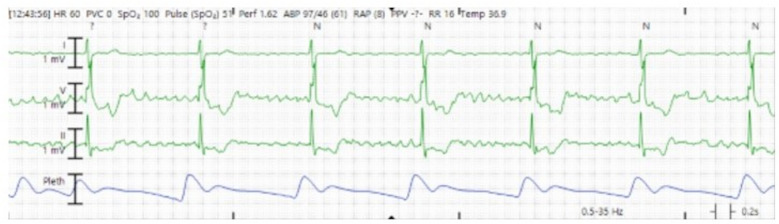
Cardiac telemetry showing underlying junctional escape rhythm consistent with atrioventricular block.

The patient's clinical course was notable for tachycardia-induced cardiomyopathy requiring ongoing hemodynamic support with epinephrine, vasopressin, and norepinephrine. Again, his atrial flutter was refractory to repeat cardioversion as well as medical therapy including esmolol, diltiazem, and digoxin. Ultimately, in part due to abnormal cardiac positioning and limited vascular access options (due to peripheral VA ECMO support), three attempts at AV nodal ablations, a temporary pacemaker, and subsequent permanent pacemaker insertion were needed for his refractory atrial flutter. During the third attempt, a radiofrequency ablation catheter was advanced via left femoral venous access into the right ventricle, where the His potential was identified. The catheter was then repositioned to a more proximal site with a small His potential, and radiofrequency energy was applied at 30–40 W, resulting in a complete heart block with a junctional escape rhythm at 44 bpm (Figure 4). Additional ablation lesions were applied, and by the procedure's conclusion, no escape rhythm was identified at a pacemaker setting of VVI 30 bpm. He required ECMO for 10 days and was successfully decannulated following rate control with AV nodal ablation, ventricular pacing, and recovery of left ventricular function (LVEF 55%).

Due to recurrent aspiration events during attempted extubation from mechanical ventilation, the patient developed aspiration pneumonia. He was treated with cefepime, vancomycin, and metronidazole, which was de-escalated to levofloxacin upon speciation of *Klebsiella* pneumoniae. The etiology of these extubation failure events was likely due to ICU myopathy. The patient ultimately underwent tracheostomy placement due to chronic hypercarbic respiratory failure.

The remainder of the patient's hospital course was significant for iatrogenicity as well as the progression of his underlying disease. He continued to have intermittent atrial flutter with an HR of <100, due to AV discordance. Additionally, he had persistent systolic dysfunction (LVEF 23%–30%) despite milrinone, likely due to the loss of AV synchrony. He was determined not to be a candidate for advanced heart failure therapies such as a ventricular assist device (VAD) or heart transplant. Particular considerations were due to his severe scoliosis, small left ventricular cavity, mixed cardiomyopathy physiology, and the high likelihood of needing biventricular support. Moreover, due to his comorbidities (tracheostomy–ventilator dependence, non-ambulatory baseline, delirium, physical deconditioning), the potential for engagement in rehabilitation status post VAD or heart transplant would be significantly limited.

Ultimately, although the patient's arrhythmia burden was controlled, his systolic function did not recover. The multisystem clinical manifestations of his FRDA had a significant impact on his cardiac and critical care management. Specifically, his severe rotatory neuromuscular scoliosis and his resultant abnormal thoracic cavity had a notable impact on the management options available for his cardiac arrhythmias and heart failure. After interdisciplinary input from multiple pediatric subspecialty teams including critical care, cardiology, neurology, and palliative medicine, the patient was discharged home with supportive care. The patient's family declined hospice referral.

## Discussion

FRDA is the most common hereditary ataxia, occurring in approximately 1 in 50,000 individuals (3). The disease severity is correlated with both young age of onset and longer length of GAA repeat expansions (3–5). Thus, the presence of severe disease affecting multiple organ systems in young patients, similar to this case, has great relevance in pediatric critical care.

The literature describes a wide range of GAA repeat expansions, typically spanning 120–1,700 (3). Consistent with this, the patient's GAA repeat expansion of 900 was associated with early disease onset, severe clinical manifestations, and rapid progression (3–5). Notably, studies have also linked longer GAA repeat expansions to a higher frequency of hypertrophic cardiomyopathy, further emphasizing the importance of genetic factors in disease expression and management (3, 14).

Of note, the patient's uncorrected neuromuscular scoliosis substantially limited management options. The patient was scheduled to undergo posterior spinal fusion surgery 18 months prior to admission, but the procedure was aborted due to undetectable neurological signals, placing him at high risk for neurological deterioration. Shortly after, a spinal MRI showed no intrathecal abnormalities. At the time, the patient retained bowel and bladder continence and could ambulate briefly for transfers. His parents subsequently opted for a comfort-directed palliative care approach. Although up to 90% of early-onset FRDA patients develop scoliosis, corrective surgery is often delayed (6). While often postponed until the end of the growth period, these patients experience rapid neurological decline and often lose ambulation during or soon after surgical correction (6).

Hypertrophic cardiomyopathy is the most common and well-recognized cardiac complication of FRDA (10). While less prevalent, atrial arrhythmias can also significantly impact these patients. Although data on the etiology of atrial and refractory arrhythmias in FRDA are limited, the prevailing hypothesis suggests that myocardial fibrosis and subsequent scarring increase the risk of arrhythmias (15). In the present case, the patient's refractory arrhythmias were largely iatrogenic, driven by AIT type 2, which exacerbated tachyarrhythmias. A literature review identified only one other reported case of AIT in FRDA: a 26-year-old patient with hypertrophic cardiomyopathy who developed intraoperative thyroid storm characterized by hyperthermia, tachycardia, and hypertension during thyroidectomy for refractory thyrotoxicosis (16). However, additional reports have linked organic thyroid dysfunction in FRDA to worsening cardiac function (17, 18). One case described persistent sinus tachycardia in a 14-year-old with FRDA (without cardiomyopathy) due to undiagnosed Graves' disease, first identified during diabetic ketoacidosis (17). Another reported a 16-year-old with FRDA (with hypertrophic cardiomyopathy) who was repeatedly hospitalized for nonspecific symptoms, including mild tachycardia, weight loss, emesis, hypovolemia, and altered mental status (18). This patient was later diagnosed with Graves' disease in the setting of severe systolic dysfunction (EF 16%), which improved to 39% after methimazole initiation (18). These cases, along with the present one, underscore the role of endocrinopathies in exacerbating cardiac dysfunction in FRDA. They also suggest a potential increased risk of thyroid disease in FRDA, highlighting the importance of routine endocrine screening (16–18).

This case highlights an unusual presentation of medication- and procedure-refractory cardiac arrhythmias, resulting in episodic acute heart failure, in an adolescent with FRDA. Definitive management was significantly impacted by the patient's atypical anatomy (uncorrected severe rotatory scoliosis). This resulted in multisystem complications (heart failure, respiratory failure, sepsis, hyperthyroidism), prolonged ICU and hospital length of stay, significant iatrogenicity (delirium, deconditioning), and high healthcare resource utilization in the setting of disease progression.

Given the progressive nature of FRDA, particularly with the patient's GAA repeat length of 900, early pediatric palliative care involvement was prioritized. As such, the patient was followed as an outpatient and throughout the hospitalization. Multiple goals of care discussions took place, with the family expressing a strong preference for all possible disease-directed and life-prolonging measures.

## Conclusion

Intensivists and cardiologists should be cognizant of the multisystem complexities involved in managing complex cardiac arrhythmias, particularly in those with concomitant genetic conditions, as demonstrated in this case of FRDA. This patient's severe rotatory scoliosis and progressive cardiomyopathy significantly altered arrhythmia management, limiting procedural success, complicating critical care interventions, and contributing to heart failure. The interplay between genetic disease, structural abnormalities, and medical therapy highlights the importance of a patient-centered, multidisciplinary approach to anticipate and mitigate iatrogenicity.

This case highlights key considerations in managing critically ill pediatric patients with genetic conditions and complex arrhythmias. First, AIT, as well as other known endocrinopathies, can worsen tachyarrhythmias and precipitate acute heart failure in FRDA, underscoring the need for routine endocrine screening. Second, proactive ICU liberation strategies can help prevent iatrogenic complications such as delirium, deconditioning, and ventilator dependence. Third, uncorrected neuromuscular scoliosis can impair baseline cardiopulmonary function and limit potential cardiac interventional options and overall outcomes. Finally, early and ongoing pediatric palliative care involvement ensures that medical interventions align with patient and family goals in the setting of progressive multisystem disease.

This case not only informs intensivists and cardiologists of the acute and subacute complications associated with refractory atrial arrhythmias in FRDA but also reinforces the broader principles of critical care—ICU liberation and de-escalation, comprehensive interprofessional care, and prioritizing quality of life in the face of progressive disease.

## Data Availability

The original contributions presented in the study are included in the article/Supplementary Material, further inquiries can be directed to the corresponding author.
